# LeafXNet: A Lightweight Explainable Model With Integrated Attention Mechanism for Fruit Leaf Disease Detection

**DOI:** 10.1002/pld3.70201

**Published:** 2026-10-22

**Authors:** Shah Murtaza Rashid Al Masud, Farhana Sultana Eshita, Abu Bakar Siddique Mahi, Tasnim Jahin Mowla, Noor Mairukh Khan Arnob, Aloke Kumar Saha, M. F. Mridha

**Affiliations:** ^1^ Department of Computer Science and Engineering University of Asia Pacific Dhaka Bangladesh; ^2^ BUBT Research Graduate School Bangladesh University of Business and Technology Dhaka Bangladesh; ^3^ Department of Computer Science and Engineering American International University of Bangladesh Dhaka Bangladesh

**Keywords:** agricultural AI, deep learning, explainable AI, fruit leaf disease detection, Internet of Things, self‐attention mechanism

## Abstract

Food security is threatened by climate change, altered precipitation patterns, soil degradation, and overuse of pesticides. These challenges compromise the sustainability of agricultural practices and increase the financial burden on farmers. Fruits such as watermelons, grapes, and guava, which are rich in nutrients, play a crucial role in global diets and economies. However, their production often faces challenges owing to emerging diseases and inadequate detection techniques. Conventional approaches to fruit leaf disease management remain inadequate, particularly in settings with limited resources. Consequently, there is a pressing need for reliable, economical, and scalable detection systems to safeguard agricultural outputs and improve global food security. To address these challenges, we introduced LeafXNet, a lightweight attention‐enhanced convolutional neural network (CNN) with only 79,717 parameters for fruit leaf disease detection. It demonstrates effective generalization and resource‐efficient performance suitable for web, mobile, and Internet of Things applications. We integrate three distinct methods of Explainable AI (XAI) to enhance understanding and foster trust in the predictions made by the model. The results demonstrate that LeafXNet surpasses state‐of‐the‐art pretrained CNN architectures while utilizing substantially fewer computational resources, achieving accuracies of 99.74 ± 0.0032, 99.76 ± 0.0029, and 97.59 ± 0.0086 for watermelon, grape, and guava leaf disease classification, respectively. However, the model is limited to disease‐versus‐healthy classification and requires validation on visually similar nondisease conditions before real‐world deployment.

## 1 Introduction

Worldwide food security is a crucial concern, because forecasts indicate that food production will need to double by 2050 to fulfill the demands of the rising worldwide population (Nations 2009). Food security is determined not only by the availability of calories but also by access to healthy foods. Expanding the production of nutrient‐rich crops, such as fruits, helps stabilize the food system and improve health outcomes. Climate change and global warming pose substantial concerns to agriculture, affecting crop production and insect behavior globally (Skendžic´ et al. 2021). Increasing temperatures, changing precipitation patterns, and higher CO_2_ concentrations have hastened the spread and survival of insect pests, increased insect‐borne plant diseases, and impaired natural biological controls. These changes increase the possibility of crop failure and threaten global food security, highlighting the critical need for improved plant disease detection and control measures. Several internationally cultivated and widely consumed fruits, such as watermelon, guava, and grape, are both popular and nutritious. However, their increased susceptibility to various ailments poses a considerable danger to output, quality, and market value. Watermelon is one of the most widely farmed fruits in the world, with China producing over 73 million tons each year, or more than 60% of the total (Kaur 2025). Watermelon is one of the top five fruits in terms of volume, with more than 100 million tons produced worldwide. Grapes drive the global wine business by producing approximately 75 million tons annually (Kumar 2024). The guava industry is expected to increase from $20.46 billion in 2025 to $37.40 billion by 2034 (Tyagi and Anand 2025).

Numerous studies have developed accurate prediction models for the early identification of fruit leaf disease. Early detection reduces crop damage and allows early action to reduce losses. Some studies have applied deep metric learning (DML) with triplet loss to classify watermelon export varieties (Contreras et al. 2022). However, this study encountered challenges with class imbalance and feature overlap, particularly in distinguishing one particular variety, which led to overfitting and misclassification. Another study (Abdullah et al. 2012) suggested a fuzzy‐logic–based classification method to identify watermelon leaf diseases utilizing RGB color data derived from 200 leaf samples, attaining above 67% accuracy for both diseases. Despite its comparatively poor classification accuracy, the proposed fuzzy logic model may not yield reliable illness detection for practical applications. Thet et al. (2020) optimized Visual Geometry Group 16‐layer model (VGG16) by substituting its completely linked layers with a global average pooling (GAP) layer to identify five predominant grape leaf diseases, with an accuracy of 98.4%. However, the study did not optimize epochs and batch size, which affected the overall performance. Nagaraju et al. (2020) optimized a pretrained VGG16 model via Keras to categorize eight diseases affecting apple and grape leaves, attaining an accuracy of 97.87% with an 80:20 dataset division. The study exhibited robust performance, although minimal enhancement was observed after training for 30 epochs. Almadhor et al. (2021a) created an AI‐based model to identify four diseases affecting guava leaves and fruits by employing five classifiers, with the bagged tree attaining the best accuracy of 99%. However, this methodology was constrained by its modest dataset of only 393 images. Perumal et al. (2021) introduced an SVM‐based approach aimed at identifying a specific guava leaf disease and achieved an accuracy of 98.17%.

Recent studies have highlighted numerous challenges that hinder the precise detection and classification of diseases that affect fruit plants. The challenges include low image contrast, inconsistencies in the shape, texture, and size of infected areas, and uneven lighting conditions. Additionally, numerous employed models depend on excessively complex architectures and function as black‐box systems, which further restrict their interpretability and real‐world usability. This study presents a new method for identifying diseases in fruit plants, with a particular emphasis on watermelon, grape, and guava, with the aim of overcoming the existing challenges. The aim is to enhance the precision in classifying healthy and diseased leaves. The study presents its key contributions as outlined below:

- We propose LeafXNet, a lightweight, attention‐fused Convolutional Neural Network (CNN) with only 79,717 parameters, specifically designed for fruit leaf disease detection and optimized for deployment in IoT, mobile, and web applications.
- We evaluated our lightweight model on three distinct datasets, unlike previous approaches limited to a single fruit leaf disease, demonstrating a strong generalization capability and resource‐efficient performance.
- We enhance the interpretability of our model by incorporating three distinct Explainable AI (XAI) techniques, in contrast to traditional black‐box approaches, thereby promoting transparency and enabling greater user trust.
- We compared our work with recent research works, and a comprehensive analysis demonstrated that LeafXNet outperforms state‐of‐the‐art pretrained CNNs, achieving higher accuracy in fruit leaf disease detection while using fewer computational resources.

The paper is structured as follows:

Section 2 offers a comprehensive overview of prior research in the domain. Section 3 provides a thorough explanation of the proposed technique. The Section 4 provides a comprehensive examination of the experimental findings. In conclusion, the paper summarizes with Section 5.

## 2 Related Works

### 2.1 Watermelon Disease Detection

Extensive research has been undertaken over the years on precise watermelon fruit categorization and disease detection, with the aim of improving crop management methods and reducing the risk of disease outbreaks. Ganatra and Patel (2020) introduced an automated model for the detection and classification of plant leaf diseases via image processing and machine learning methodologies, including random forest, SVM, KNN, and ANN. The random forest classifier attained a maximum accuracy of 73.38% on a dataset including 14,956 pictures across 38 illness categories. The methodology encompasses preprocessing, segmentation, and feature extraction focusing on the form, color, texture, and vein patterns. Nonetheless, the model's efficacy declines with an increase in the number of classes and photos, underscoring the increasing challenges and the necessity for more refined algorithms to manage larger and more complex datasets. Jayakumar et al. (2020) introduced a stacked recurrent neural network (RNN)–based deep learning model for automated prediction and categorization of illnesses in melon plants. This methodology incorporates picture preprocessing, segmentation by K‐means clustering, feature extraction, and classification through the stacked RNN model, exhibiting superior accuracy and reduced execution time relative to traditional techniques. The study has gaps in extensive performance evaluation, failing to include full accuracy values or additional important metrics such as precision, recall, and F1‐score. To accurately detect and classify watermelon disorders, another study by Islam et al. Islam et al. (2024) developed a hybrid deep learning model called Dense‐EfficientNet, which combines DenseNet121 and EfficientNetV2B0. The model further enhances interpretability by the using of LIME and Grad‐CAM approaches, and it attained a high accuracy of 98.40% with great precision, recall, and F1‐scores. However, the model's size and complexity make it unsuitable for deployment on edge devices with limited resources, such as smartphones or Internet of Things (IoT) sensors, even though it is accurate.

Tas et al. (2025) introduced a method that employs CNN to detect watermelons in aerial images captured by unmanned aerial vehicles (UAVs). The model employed real‐world field data and demonstrated the effectiveness of deep learning for automating fruit detection tasks in precision agriculture. However, the study encountered limitations owing to the computational complexity of the proposed model, which renders it less suitable for real‐time or practical implementation in resource‐limited agricultural settings. These elements emphasize how model accuracy and practical implementation must be balanced in agricultural environments with limited resources. Alhazmi (2023) recommended the use of the CNN VGG16 to identify diseases in watermelon plants. The model's image classification is extremely precise owing to its structure and number of layers, and the time required for model optimization and training is reduced because it has two convolutional layers with 32 and 64 filters, two max pooling layers, one flattening layer, and two fully connected dense layers, with a kernel size of 5 × 5 in the convolutional layers, which significantly reduces the number of computations compared to deeper networks like VGG‐16. Overfitting was observed in the CNN‐VGG‐16 model, resulting in a recall of 0.7576 on the validation dataset and a minimum true positive (TP) score of 7%, indicating poor generalization. By tuning key hyperparameters, particularly weight initialization and optimizer selection, the optimized model significantly improved performance, achieving a recall of 0.9394 and a minimum TP score of 98%.

Arora et al. (2024) introduced a hybrid deep learning model that integrates RNN and random forest to evaluate the severity of Watermelon mosaic virus (WMV) in watermelon crops. The algorithm attained an exceptional 99.08% accuracy in categorizing illness into five severity categories, utilizing more than 16,000 annotated photos. It exhibited strong performance in accuracy, recall, and F1‐score measures, indicating its potential as a reliable instrument for early disease control in precision agriculture. However, a disadvantage of this study is that the model is only developed to identify and assess WMV, limiting its application to other varieties of watermelon illnesses.

### 2.2 Grapes Disease Detection

A recent study by Dharrao et al. (2025) developed a high‐quality, well‐annotated dataset of 2726 images of grape leaves from Nashik, India, taken over a 2‐year period (2023–2025). The images included both healthy leaves and three main disease classes: bacterial leaf spot, powdery mildew, and downy mildew. The dataset validated with ResNet‐18 demonstrated its potential for AI‐driven disease diagnosis and sustainable grape production with a 96% classification accuracy. Huang et al. (2020) targeted diseases such as black rot, black measles, leaf blight, and phylloxera and offered four modified deep learning models for grape leaf disease detection and classification using transfer learning from VGG16, MobileNet, and AlexNet. Combining these models in an ensemble manner further improves detection and classification accuracy, indicating significant promise for managing grapevine diseases. However, this study does not include information on computational resources, which are essential for determining viability in real‐time or large‐scale applications. Some instances of these resources include the processing time and memory. The study by Nagamani et al. (2024) provides a comparative analysis of a custom CNN and the pretrained VGG16 model for classifying grape leaf diseases, assessing the influence of various optimization algorithms (Adam, Nadam, and SGD) on accuracy. The results indicated that Adam attained peak performance, providing valuable insights into the selection of optimizers for systems aimed at detecting agricultural diseases. However, an evaluation that primarily focuses on the overall accuracy while neglecting statistical validation or testing against external datasets limits the generalizability and robustness of the findings.

Padol and Yadav (2016) developed an image processing‐based framework for the detection and classification of grape leaf diseases by employing an SVM classifier. The approach first utilized K‐means clustering to segment the diseased regions, followed by the extraction of both color and texture features, and finally applied SVM classification to identify the specific disease, achieving an accuracy of 88.89%. However, the study has several limitations: It addresses only a limited number of grape leaf diseases, demonstrates comparatively low detection accuracy, and does not incorporate comprehensive feature extraction to capture all relevant image characteristics. It also lacks a systematic feature selection process to identify the most discriminative attributes and excludes several prevalent grape leaf diseases from its analysis.

Jaisakthi et al. (2019) presented a novel system for the automatic detection of grapevine diseases, utilizing advanced image processing and machine learning methodologies in their research. The methodology comprised segmenting the leaf area from the background using the grab‐cut technique, identifying diseased regions through global thresholding and semisupervised methods, and categorizing them into healthy, rot, esca, and leaf blight classifications using SVM, AdaBoost, and random forest classifiers, with SVM achieving an accuracy of 93%. However, this approach is constrained as hand‐crafted features may fail to capture complex disease‐related variations. An automated computer vision‐based method for identifying Grapevine Measles (Esca) from images of grape leaves was presented by Singh et al. (2020). With a 97.2% accuracy rate, the approach consists of multichannel background separation, illness region segmentation using global thresholding and morphological procedures, statistical feature extraction, and classification using an SVM. The limited number of disease categories taken into consideration, however, limits thorough feature extraction and prevents the identification of the most discriminative features. The research by Lauguico et al. (2020) benefits the field of grape leaf disease detection through the development of a system based on RCNN technology. It effectively identifies three significant diseases: black rot, black measles, and Isariopsis, while also recognizing healthy leaves and incorporating confidence scores into the classification process. An examination of AlexNet, GoogLeNet, and ResNet‐18 as backbone networks demonstrated that the combination of AlexNet with RCNN attained the highest accuracy of 95.65%, surpassing the performance of the other two models. This study emphasizes the capabilities of deep learning to achieve accurate disease identification through the use of annotated images and bounding boxes. Despite this, a significant drawback is the dependence on a black‐box methodology, which is labor‐intensive and lacks clarity, as it does not explicitly identify or optimize the most effective features for disease classification.

### 2.3 Guava Disease Detection

Al Haque et al. (2019) presented a CNN‐based deep learning methodology for identifying guava fruit diseases, namely, fruit canker, anthracnose, and fruit rot, attaining an accuracy of 95.61% in studies performed in Bangladesh. The model showed significant efficacy for categorizing individual diseases. However, the inability of the model to detect multiple diseases simultaneously present on the same leaf constrained its effectiveness. Howlader et al. (2019) designed the first deep CNN‐based model for the classification of guava leaf diseases, achieving a remarkable accuracy of 98.74%. Their research presented a dataset consisting of four categories, rust, algal leaf spot, whitefly, and healthy leaves, thus providing a significant standard for future investigations of guava disease identification. Ray et al. (2025) presented an SVM‐based model for the detection of guava leaf illnesses, employing LBP, GLCM, and HSV histogram characteristics to identify four predominant diseases along with healthy leaves. The RBF kernel SVM attained a significantly superior accuracy of 91.67% compared to the linear kernel, demonstrating its efficacy for nonlinear datasets. The study is constrained by its single‐label classification, which neglects instances with numerous concurrent disorders, and the lack of pruning approaches to enhance the computational efficiency.

Almadhor et al. (2021b) developed an AI model to identify four diseases affecting guava leaves and fruits, assessing five classifiers: cubic SVM, bagged tree, fine KNN, boosted tree, and complicated tree, with the bagged tree attaining the highest accuracy of 99%. This study demonstrated the efficacy of ensemble learning in disease diagnosis. However, its primary weakness is the limited dataset of only 393 photos, which may affect the generalizability of the model. Mostafa et al. (2021) provided a deep convolutional neural network (DCNN)–based method for guava disease diagnosis, using color‐histogram equalization, unsharp masking, and significant data augmentation to enhance the classification accuracy. The approach was assessed using a locally sourced dataset from Pakistan, using various state‐of‐the‐art networks, where ResNet101 attained the highest performance at 97.74% accuracy. Nath Nandi et al. (2022) employing a dataset of 7000 photos containing 25 plant categories and achieved a maximum accuracy of 99.53% with the use of several DCNN architectures. The authors proposed suitability of the model for real‐time identification of plant diseases.

## 3 Materials and Methods

The study begins with a description of the dataset, including its sources and characteristics. To improve the robustness of the model, data augmentation techniques were applied to increase the diversity of the dataset. The proposed model architecture is then presented, highlighting its design and optimization strategies. Finally, the explainability tools used to interpret the model predictions and enhance transparency are described.

### 3.1 Dataset Description

Three distinct fruit leaf disease datasets, namely, grape, watermelon, and guava were implemented. The dataset of watermelon leaves was obtained from the Regional Horticulture Research Station in Lebukhali, Patuakhali, Bangladesh (Nakib and Mridha 2024). It includes 1155 original images of healthy leaves, as well as three varieties of leaf diseases: mosaic virus, downy mildew (caused by *Pseudoperonospora cubensis*), and anthracnose (caused by *Colletotrichum orbiculare*). To improve the generalizability of the model, data augmentation techniques were implemented, including horizontal flipping, scaling, luminance adjustment, and random zooming, resulting in a dataset of 6930 images. Downy mildew (caused by *Plasmopara viticola*), powdery mildew (caused by *Erysiphe necator*), bacterial leaf spot (caused by *Xanthomonas campestris* ), and healthy leaves comprise the grape leaf dataset (Dharrao et al. 2025), which comprises 2726 images that are collected and categorized. Domain specialists from the Grape Research Centre, Pimpalgaon Baswant, Nashik District, Maharashtra, India, provided assistance in the annotation process. The dataset exhibits a significant class imbalance between healthy and diseased images. To mitigate this issue, several data augmentation techniques were applied to the diseased class, including horizontal and vertical flipping, rotation (30°–45°), luminance adjustment, and random zooming. These transformations increase data diversity and improve model generalization.

The guava leaf disease dataset (Shihab et al. 2025) was obtained from three locations in Bangladesh: Rampur in Cumilla, Asulia Bazar in Dhaka, and Baipayl in Dhaka. It comprises 2313 original images that are categorized into five categories: anthracnose (caused by *Colletotrichum gloeosporioides*), dot (caused by *Pestalotiopsis psidii*), canker (caused by *Lasiodiplodia theobromae*), rust (caused by *Puccinia psidii*), and healthy leaf. Horizontal flipping, brightness and contrast adjustments, random rotation up to 40°, Gaussian blur with kernel size between 3 and 7, random scaling within 20%, and random application of embossing or sharpening are implemented to enhance robustness and variability. The dataset was increased to 12,262 images as a result of these augmentations. However, due to a significant class imbalance with an overrepresentation of healthy samples, a random subsampling strategy was applied to the healthy class. Specifically, a subset of healthy images was randomly selected to match the size of the smallest class (rust), thereby ensuring a more balanced class distribution for training. The datasets were randomly shuffled and then split into training, validation, and test sets using an 80:10:10 ratio. Sample instances from each class are illustrated in Figure 1, and a class‐wise summary of all datasets is provided in Table 1.

**FIGURE 1:**
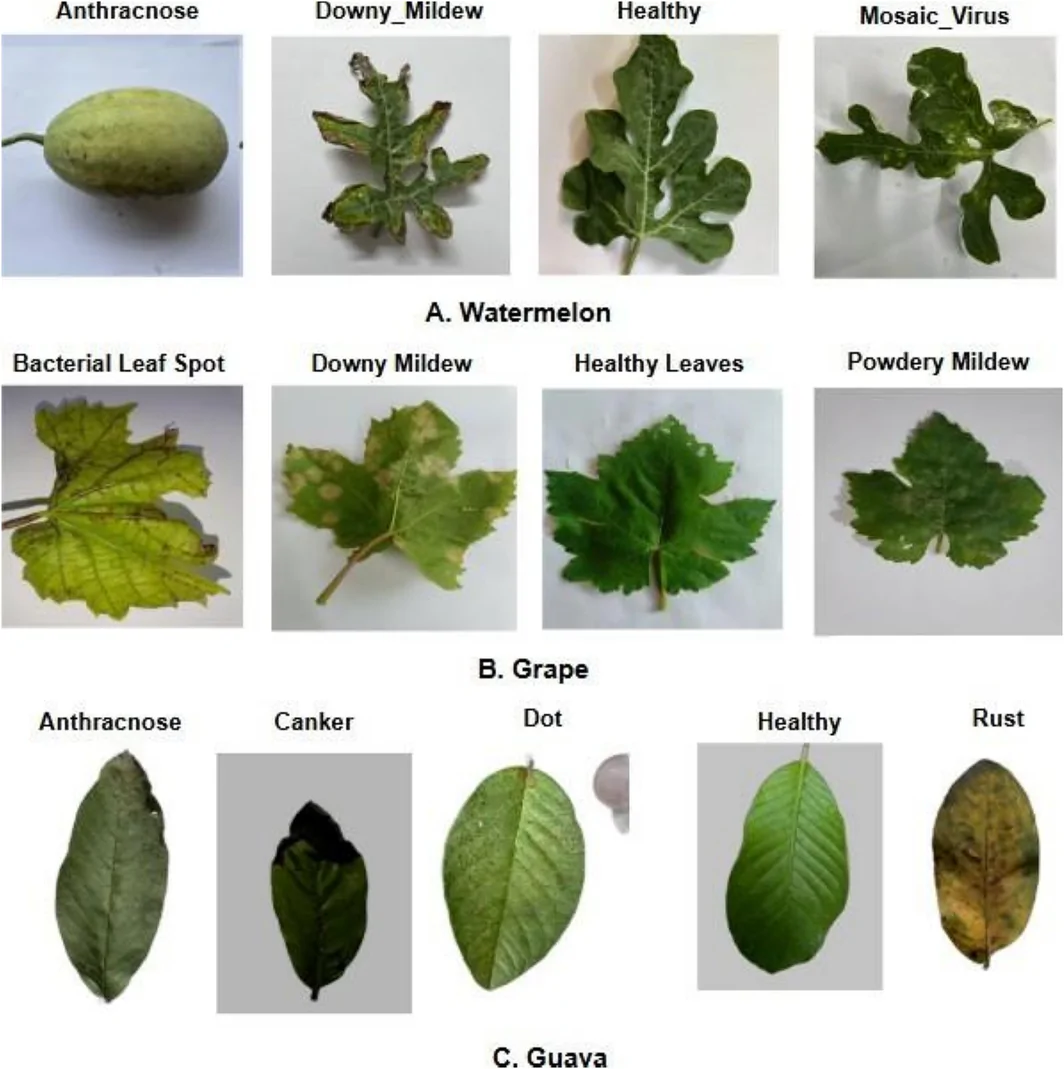
Sample fruit leaf disease data instances: (a) watermelon leaf diseases, (b) grapes leaf diseases, and (c) guava leaf diseases.

**TABLE 1:** Summary of the experimental datasets.

Data	Class names	No. of images
Watermelon	Mosaic virus Downy mildew Anthracnose Healthy	2490 2280 930 1230
	**Total**	**6930**
Grapes	Bacterial leaf spot	1254
	Downy mildew	1254
	Healthy leaves	1254
	Powdery mildew	1254
	**Total**	**5016**
Guava	Anthracnose Canker Dot	1896 1536 1752
	Healthy	1336
	Rust	1336
	**Total**	**7856**

### 3.2 Proposed Model

The LeafXNet model, as shown in Figure 2, is a lightweight, attention‐enhanced CNN (Siddique Mahi et al. 2024; Mahi et al. 2024) developed for detecting leaf diseases from RGB images of size (224, 224, 3). LeafXNet integrates convolutional feature extraction and multihead self‐attention to effectively capture the spatial and contextual attributes essential for identifying leaf diseases. The network starts with an InputLayer accepting images of shape (224, 224, 3). A Rescaling layer normalizes the pixel values to the range [0, 1], ensuring stable and efficient training. The first convolutional layer applied 64 filters of size 3 × 3, producing a feature map of size (222, 222, 64).

**FIGURE 2:**
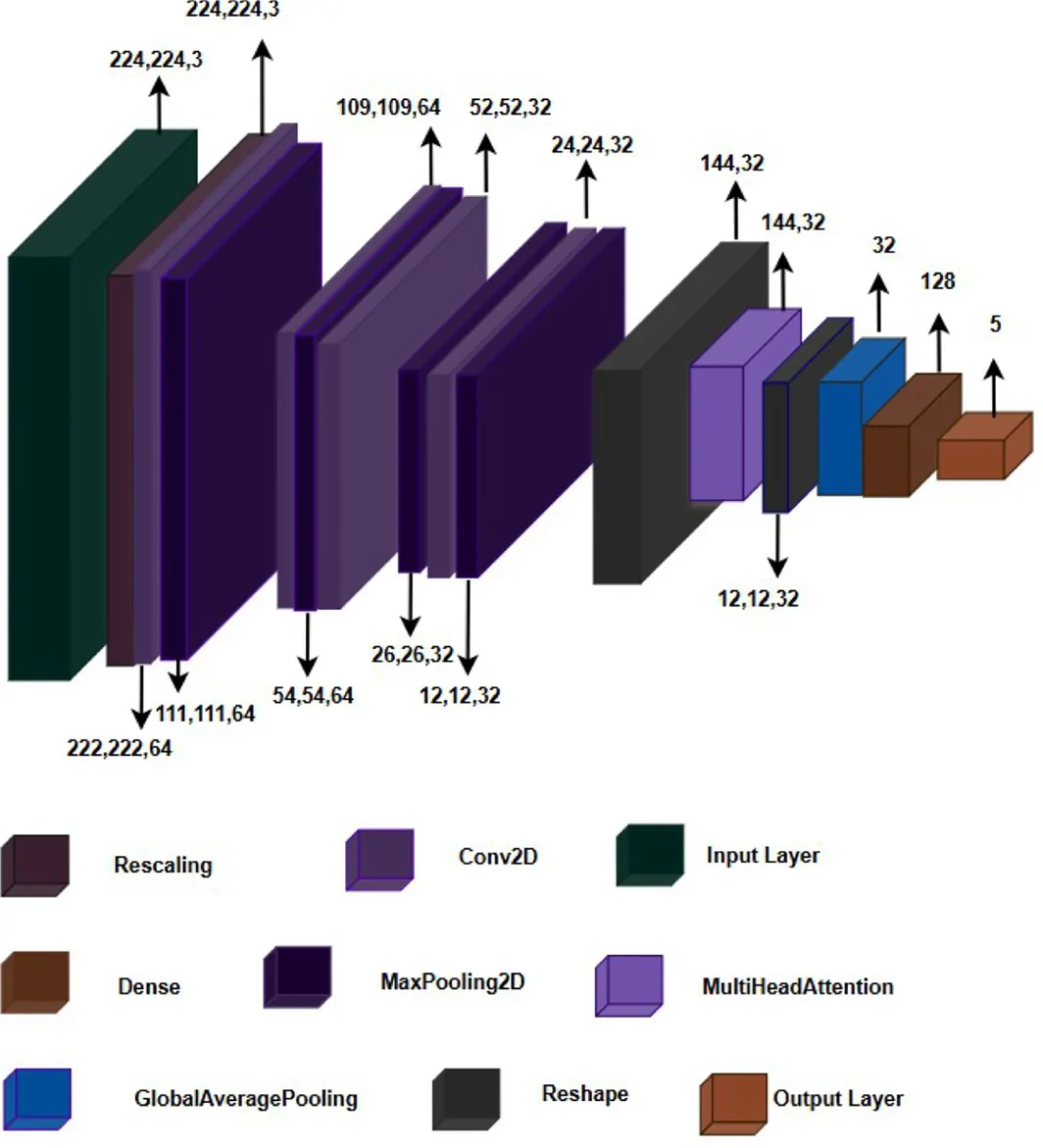
Proposed lightweight LeafXNet architecture for effective fruit leaf disease recognition.

The output was passed through a Rectified Linear Unit (ReLU) activation function. A MaxPooling2D layer with a 2 × 2 kernel reduces the feature map to (111, 111, 64). The second convolutional layer with 64 filters produced a feature map of size (109, 109, 64), followed by MaxPooling2D reducing it to (54, 54, 64). The third convolutional layer had 32 filter outputs (52, 52, 32), followed by MaxPooling2D to (26, 26, 32). Fourth convolutional layer with 32 filters produces (24, 24, 32), followed by MaxPooling2D to (12, 12, 32). For capturing long‐range dependencies and emphasizing disease‐relevant regions, the feature maps are reshaped to a sequence of shape (144, 32) and passed through a multihead attention (MHA) layer (Tonmoy et al. 2025). Attention weights are computed using queries (*Q*), keys (*K*), and values (*V*) as shown in Equation (1):

(1) $$\text{Attention}\left(Q,K,V\right)=\text{Softmax}\;\left(\frac{QK^{T}}{\sqrt{d_{k}}}\right)\cdot V$$

Multiple attention heads operate in parallel, and their outputs are concatenated and linearly projected as shown in Equation (2):

(2) $$\text{MultiHead}\left(Q,K,V\right)=\text{Concat}\left({\text{head}}_{1},\ldots ,{\text{head}}_{h}\right)\cdot W^{0}$$

The attention‐enhanced features are reshaped back to (12, 12, 32), preserving the spatial layout but enriched with context‐aware importance scores. This enables the network to focus on regions that are most indicative of disease presence while suppressing irrelevant background information. The output is then passed through a GAP layer, reducing each feature map to a single scalar by averaging across spatial locations. The GAP output was fed into a dense layer of 128 neurons to learn the high‐level abstract features. Finally, a Dense output layer with five neurons and a softmax activation function provided class probabilities for the five leaf disease categories. LeafXNet comprises 79,717 trainable parameters and combines efficient convolutional feature extraction with attention mechanisms to precisely identify disease‐specific patterns. Employing MHA allows the model to concentrate on the most significant areas within leaf images, enhancing classification accuracy and maintaining its applicability for real‐time use. Finally, Table 2 presents the architectural details of the proposed model.

**TABLE 2:** Architectural details and parameter summary of the proposed LeafXNet model.

Layer (type)	Output shape	Param #
Input layer	(224, 224, 3)	0
Rescaling	(224, 224, 3)	0
Conv2D	(222, 222, 64)	1792
MaxPooling2D	(111, 111, 64)	0
Conv2D	(109, 109, 64)	36,928
MaxPooling2D	(54, 54, 64)	0
Conv2D	(52, 52, 32)	18,464
MaxPooling2D	(26, 26, 32)	0
Conv2D	(24, 24, 32)	9248
MaxPooling2D	(12, 12, 32)	0
Reshape	(144,32)	0
MultiHeadAttention	(144,32)	8416
Reshape	(12, 12, 32)	0
GlobalAveragePooling2D	(32)	0
Dense	(128)	4224
Dense	(5)	645
Total params		79,717
Trainable params		79,717
Nontrainable params		0

### 3.3 XAI

The ability to understand black‐box models is crucial for increasing trust in automated agricultural systems (Mallinger et al. 2024), particularly as deep learning becomes more prevalent in the detection of plant and crop diseases. Three XAI techniques, Grad‐CAM, Grad‐CAM++, and class activation map (CAM), are employed to demonstrate the decision‐making process of the proposed fruit leaf disease detection model (Prabhu et al. 2025; Mengesha and Mengistie 2025). This method produces a CAM that emphasizes the areas of the leaf that are most significant in the model's prediction, clearly illustrating the visual patterns associated with the disease.

#### 3.3.1 CAM

CAM is used by CNNs to show the parts of a picture that have the greatest effect on the model's prediction. CAM offers a visual depiction of regions that substantially influence the network's classification decisions. It specifically delineates the visual regions that a CNN uses to identify a specific class. This method can be utilized with a trained classification network comprising a feature extraction backbone, GAP layer, and linear classification layer. The classification layer's class‐specific weights are multiplied by the feature maps from the network's final convolutional layer to produce the activation map (Zhou et al. 2016).

Formally, CAMs are computed using the final convolutional feature map $\Phi \in {\mathbb{R}}^{H\times W\times D}$, where *H* and *W* denote the spatial dimensions and *D* is the number of channels. The CAM for a particular class γ, denoted as $M_{\gamma }$, is obtained as follows:

(3) $$M_{\gamma }=\sum_{d=1}^{D}\omega_{\mathrm{\gamma d}}\cdot \Phi_{d}$$

As shown in Equation (3), $\omega_{\mathrm{\gamma d}}$ represents the weight in the fully connected layer corresponding to class γ and channel *d*, whereas $\Phi_{d}$ denotes the $d^{\mathrm{th}}$ channel of the feature map Φ.

#### 3.3.2 Grad‐CAM

Grad‐CAM uses gradients from the last convolutional layer of a machine learning model to provide visual explanations (Selvaraju et al. 2017). To explain this, the gradients of the score for class c were calculated based on the activations of the feature maps in a certain convolutional layer. Here, $S^{c}$ denotes the model's pre‐softmax score for class c, which corresponds to the raw output used to determine the model's prediction. The gradients of $S^{c}$ with respect to each activation map $F^{k}$, where $F^{k}$ is the $k^{\mathrm{th}}$ feature map in the final convolutional layer, are then calculated. These gradients show how much each point in the feature map affects the prediction for class c. After that, GAP was used on these gradients to determine the important weights for each feature map.

(4) $$w_{k}^{c}=\frac{1}{N}\sum_{m}\sum_{n}\frac{\partial S^{c}}{\partial F_{m,n}^{k}}$$

In Equation (4), $w_{k}^{c}$ is the importance weight for feature map k with respect to class c. N is the total number of spatial locations in the feature map. $F_{m,n}^{k}$ is the activation at spatial location $\left(m,n\right)$ in the $k^{\mathrm{th}}$ feature map. $\frac{\partial S^{c}}{\partial F_{m,n}^{k}}$ is the gradient of the class score with respect to the activation.

#### 3.3.3 Grad‐CAM++

Grad‐CAM++ extends the original CAM by incorporating pixel‐level gradient information together with techniques such as guided backpropagation and deconvolution (Chattopadhay et al. 2018; Hasan et al. 2026). Unlike the standard CAM, higher order derivatives are used to accurately estimate the pixel contributions, thereby producing more reliable class‐specific activation maps. The confidence score for a given class *c*, denoted as $S^{\left(c\right)}$, is expressed in Equation (5):

(5) $$S^{\left(c\right)}=\sum_{m}\beta_{m}^{\left(c\right)}\cdot \sum_{p}\sum_{q}F_{\mathrm{pq}}^{m}$$

where $S^{\left(c\right)}$ represents the prediction score of class c, and $F_{\mathrm{pq}}^{m}$ corresponds to the activation at the spatial position $\left(p,q\right)$ of the $m^{\mathrm{th}}$ feature map. The class‐specific weight coefficient $\beta_{m}^{\left(c\right)}$ was obtained as follows:

(6) $$\beta_{m}^{\left(c\right)}=\sum_{p}\sum_{q}\gamma_{\mathrm{pq}}^{\left(m,c\right)}\cdot \text{ReLU}\left(\frac{\partial S^{\left(c\right)}}{\partial F_{\mathrm{pq}}^{m}}\right)$$

In Equation (6), $\gamma_{\mathrm{pq}}^{\left(m,c\right)}$ denotes the neuron importance factor at location $\left(p,q\right)$. The ReLU operator ensures that only positive gradient contributions are considered. Finally, the class‐discriminative localization map, $M_{\mathrm{pq}}^{\left(c\right)}$, is defined as in Equation (7):

(7) $$M_{\mathrm{pq}}^{\left(c\right)}=\text{ReLU}\left(\sum_{m}\beta_{m}^{\left(c\right)}\cdot F_{\mathrm{pq}}^{m}\right)$$

### 3.4 Experimental Setup

The experiments were conducted in the authors research lab using a workstation equipped with an Intel Core i7 (13th Generation) processor, 64‐GB RAM, 256‐GB SSD, 2‐TB HDD, and NVIDIA RTX 4080 GPU with 16‐GB memory. The proposed model was implemented in Python with the support of Scikit‐learn, TensorFlow, Keras, and neural network libraries. For evaluation, each dataset was partitioned into training, validation, and testing subsets in the ratio of 80%, 10%, and 10%. Table 3 summarizes the experimental settings.

**TABLE 3:** Summary of the model training settings.

Attribute	Value
Image size	224 *×* 224
Batch size	32
Learning rate	1 *×* 10^−3^
Max epoch	50
Attention head	2
Activation	ReLU
Optimizer	Adam
Loss	Sparse Categorical crossentropy
Rescale factor	1.0/255

### 3.5 Evaluation Metrics

The confusion matrix contrasts predicted outcomes with actual class labels, clearly presenting TP, true negatives (TN), false positives (FP), and false negatives (FN). Classification models are typically evaluated using a set of performance metrics derived from the confusion matrix, such as accuracy, precision, recall, and F1‐score. These confusion matrix–based metrics provide a systematic and quantitative assessment of the effectiveness of classification algorithms. In this study, we evaluated the efficacy of our suggested model using measures, including recall, accuracy, precision, and F1 score. All of these metrics are based on the main ideas mentioned above. We have also computed macro‐average and micro‐average to evaluate the overall performance of our framework.

(8) $${\bar{M}}_{\text{macro}}=\frac{1}{C}\sum_{j=1}^{C}M_{j}$$

As presented in Equation (8), the macro‐average computes the metric for each class independently and subsequently determines the arithmetic mean over all classes, treating each class with equal weight irrespective of its frequency.

(9) $${\bar{M}}_{\text{weighted}}=\frac{1}{N}\sum_{j=1}^{C}\left(M_{j}\times n_{j}\right)$$

Here, ${\bar{M}}_{\text{macro}}$: macro‐averaged metric, ${\bar{M}}_{\text{weighted}}$: weighted‐averaged metric, $M_{j}$: metric value (e.g., precision, recall, and F1‐score) for class j, C: total number of classes, N: total number of samples in the dataset, $n_{j}$: number of true instances belonging to class j (support of class j).

Conversely, the weighted average Equation (9) computes the metric for each class and subsequently averages it, weighted by the number of actual occurrences in each class, thus rendering the outcome more indicative of the dataset's class distribution.

## 4 Results

This section outlines the experimental settings and results, starting with an in‐depth evaluation of the proposed model using the training, validation, and test sets. It then presents class‐wise performance metrics and an analysis of the confusion matrix. Finally, this section presents a comparative performance evaluation of the proposed method against pretrained deep learning models.

### 4.1 Performance Evaluation

The training versus validation accuracy of the proposed architecture is presented in Figure 3 to initiate the performance evaluation, and the corresponding loss curves are depicted in Figure 4. From these figures, it is clear that the model exhibits robust generalization capabilities during the initial phases of training. The robustness of the architecture for accurately classifying leaf maladies was demonstrated by the consistent performance of the validation set over successive epochs. After training, the weights that performed best were reinstated based on the accuracy of the validation. To be more precise, the training accuracy for the watermelon disease dataset was 99.96%, whereas for grapes, it was 99.88%, and for guava, it was 97.63%. The validation accuracy of the model was 99.86% for watermelon, 100.00% for grapes, and 97.58% for guava.

**FIGURE 3:**
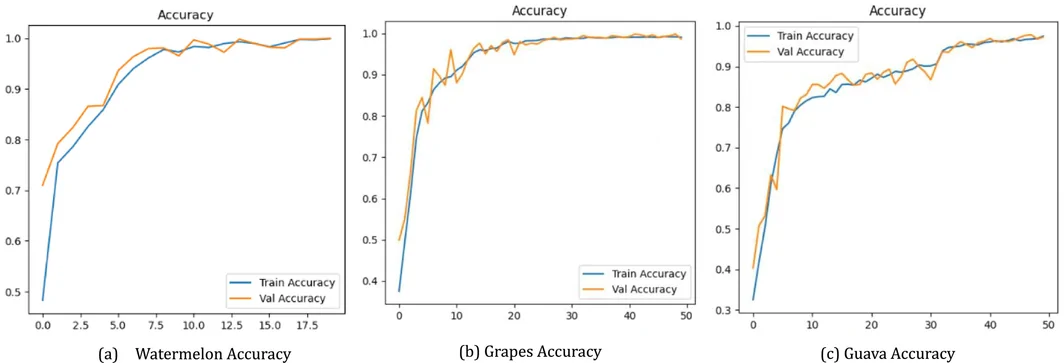
Training and validation accuracy for watermelon, grapes, and guava.

**FIGURE 4:**
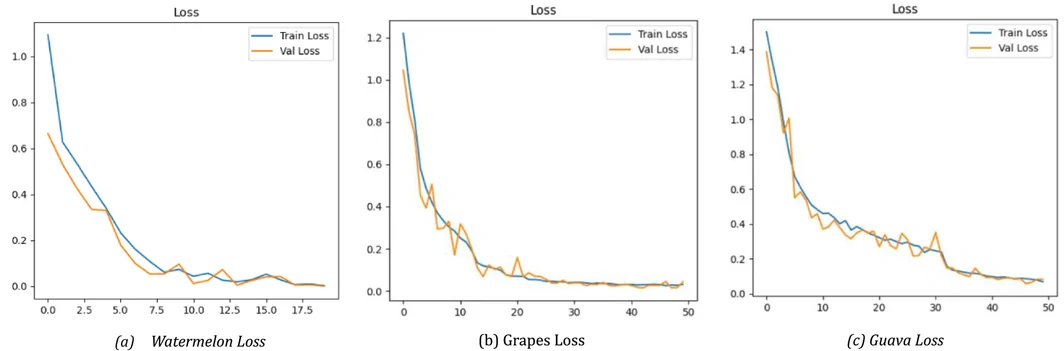
Training and validation loss for watermelon, grapes, and guava.

In addition, the proposed LeafXNet achieved superior test accuracies of 100%, 99.80%, and 97.33% for watermelon, grapes, and guava, respectively, as summarized in Table 4. The classification report in Table 5 provides a detailed analysis of the results, which are summarized in Table 4. The results indicate that the model performed exceptionally well on all three datasets, with consistently high values across multiple evaluation metrics. This report offers a thorough, class‐specific analysis of performance. LeafXNet obtained a perfect classification of watermelon leaf diseases across all classes, resulting in a 100% accuracy, precision, recall, and F1‐score. The reliability of the model for this dataset was underscored by the fact that both macro and weighted averages recorded 100% across all metrics. The model effectively identified all instances of *Bacterial Leaf Spot* and other affected leaves, achieving an overall accuracy of 99.80% for grape leaf disease classification. The class with the lowest performance was healthy leaves, with F1‐score of 99.62%.

**TABLE 4:** Performance comparison of datasets on training, validation, and test sets.

Dataset	Train acc.	Train loss	Val. acc.	Val. loss	Test acc.	Test loss
Watermelon (2024)	99.96%	0.0029	99.86%	0.0103	100%	0.0116
Grapes (2024)	99.88%	0.0041	100.00	0.0026	99.80	0.0029
Guava (2022)	97.63	0.0619	97.58	0.0598	97.33	0.0430

**TABLE 5:** Class‐wise performance summary of the proposed LeafXNet architecture over test sets.

Fruit	Class	Precision (%)	Recall (%)	F1‐score (%)	Support
Watermelon	Anthracnose	100.00	100.00	100.00	83
	Downy mildew	100.00	100.00	100.00	246
	Healthy	100.00	100.00	100.00	118
	Mosaic virus	100.00	100.00	100.00	246
	Accuracy Macro‐average	100.00	100.00	100.00 100.00	693 693
	Weighted average	100.00	100.00	100.00	693
Grapes	Bacterial leaf spot	100.00	100.00	100.00	127
	Downy mildew	100.00	99.16	99.57	118
	Healthy leaves	99.23	100.00	99.62	131
	Powdery mildew	100.00	100.00	100.00	127
	Accuracy			99.80	503
	Macro‐average	99.80	99.80	99.80	503
	Weighted average	99.80	99.80	99.80	503
Guava	Anthracnose	97.56	97.56	97.56	205
	Canker	100.00	100.00	100.00	153
	Dot	94.86	97.08	95.95	171
	Healthy	94.53	99.18	96.80	122
	Rust	100.00	92.65	96.18	136
	Accuracy			97.33	787
	Macro‐average	97.39	97.29	97.33	787
	Weighted average	97.40	97.33	97.33	787

In terms of guava leaf disease classification, the model demonstrated exceptional performance for *Rust* and *Canker*, with F1‐scores of 100.00%. *Anthracnose* and *Dot* exhibited slightly lower performances, with F1‐scores of 97.56% and 95.95%, respectively. The model achieved an overall test accuracy of 97.33%, with both the weighted‐average F1‐score and macro‐average F1‐score also reaching 97.33%. The confusion matrices for the test sets are illustrated in Figure 5 to provide a detailed visualization of the true versus deceptive predictions for each class, thereby further supporting these findings. This representation enhanced the classification report by elucidating the strengths and limitations of each class. The model accurately classified all classes in the watermelon dataset without any errors, as demonstrated. In contrast, the model demonstrated a slightly diminished ability to differentiate the *Downy Mildew* class in the context of grape leaf diseases. However, this was primarily due to the limited number of test samples, with only a single misclassified instance observed.

**FIGURE 5:**
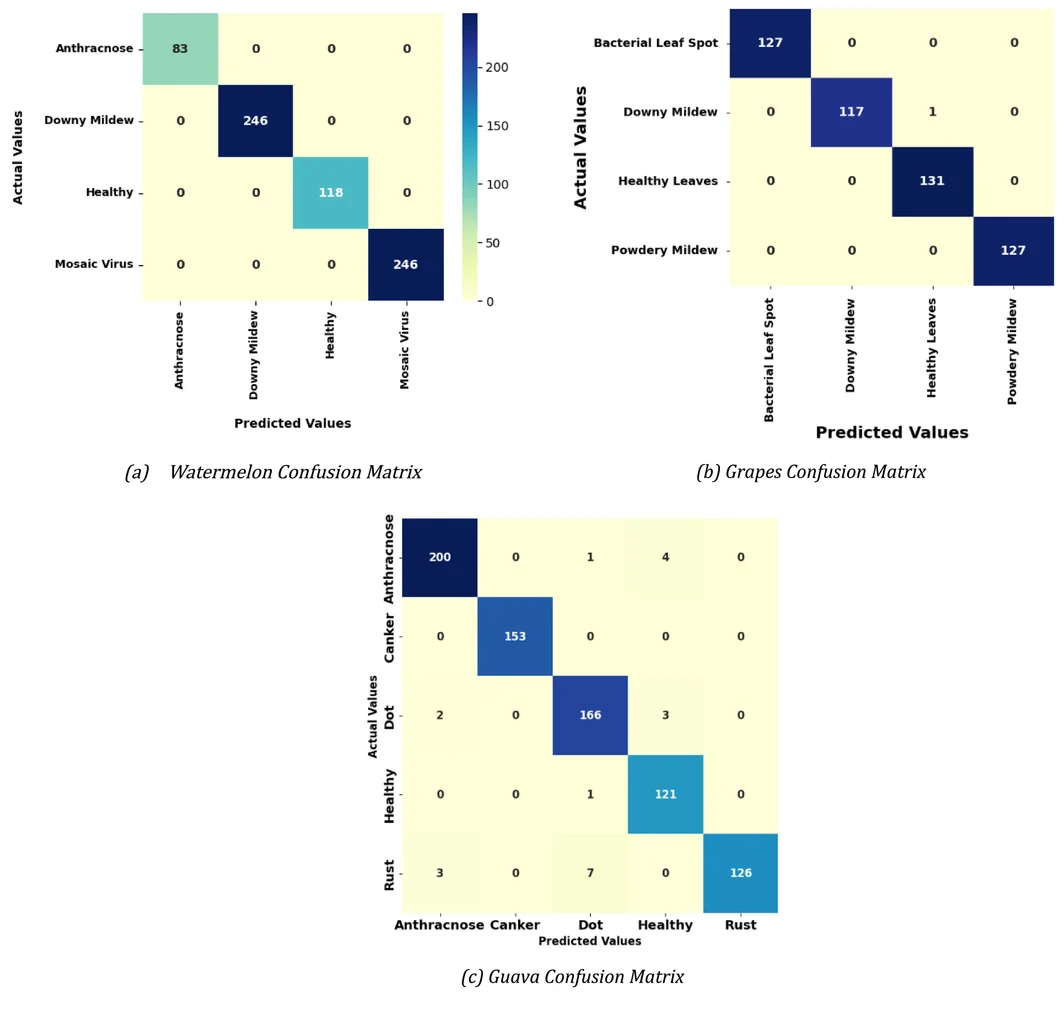
Confusion matrices for watermelon, grapes, and guava on test set.

### 4.2 Performance Comparison With Baseline Models

The proposed LeafXNet model was compared to six state‐of‐the‐art CNN architecture, Xception, DenseNet121, DenseNet169, InceptionV3, NASNetLarge, and NASNetMobile for the purpose of disease detection in watermelon, grapes, and guava, as illustrated in Table 6. LeafXNet consistently outperformed all baseline models across all evaluation metrics, achieving 100% accuracy, precision, recall, and F1‐score for watermelon, 99.80% across all metrics for grapes, and 97.33% accuracy, 97.39% precision, 97.29% recall, and 98.30% F1‐score for guava. The Xception and NASNet variants attained marginally lower accuracies between 84% and 94% for watermelon, whereas DenseNet121 and DenseNet169 performed strongly, reaching accuracies above 99%. XCEPTION and InceptionV3 continue to demonstrate the highest accuracy among the baselines for grapes. In contrast, DenseNet121, DenseNet169, NAS‐NetLarge, and NASNetMobile exhibited slightly lower performance (97%–98%). XCEPTION and DenseNet121 achieve an accuracy of approximately 81%–82% for guava, whereas the other models ranged from 51% to 77%. All baseline models exhibited significantly inferior performance. The ability of LeafXNet to achieve these results with only 79,717 parameters, as opposed to the millions in conventional CNNs, is remarkable. This demonstrates that it is both computationally efficient and highly accurate, making it an ideal choice for real‐time fruit leaf disease detection in resource‐constrained environments.

**TABLE 6:** Comparison of the performance of the proposed model with state‐of‐the‐art baseline models.

Model	Parameters	Accuracy (%)			Precision (%)			Recall (%)			F1‐score		
		W	Gr	G	W	Gr	G	W	Gr	G	W	Gr	G
Xception	21,124,397	94.43	95.94	66.75	94.55	95.97	67.39	94.43	95.94	66.75	94.42	95.93	66.26
DenseNet121	7,169,349	99.18	97.81	82.45	99.19	97.84	82.70	99.18	97.81	82.45	99.18	97.79	82.19
DenseNet169	12,856,645	99.46	97.81	80.21	99.47	97.87	80.54	99.46	97.81	80.21	99.46	97.80	78.95
InceptionV3	22,065,701	87.36	94.06	57.37	88.11	94.44	51.54	87.36	94.06	57.37	87.29	94.04	51.92
NASNetLarge	85,950,551	91.58	92.50	63.14	91.62	92.58	65.06	91.58	92.50	63.14	91.54	92.51	60.37
NASNetMobile	4,405,657	84.65	94.37	71.55	85.90	94.49	71.34	84.65	94.37	71.55	84.31	94.35	70.57
**LeafXNet**	**79,717**	**100**	**98.54**	**98.24**	**100**	**98.54**	**98.29**	**100**	**98.54**	**98.24**	**100**	**98.54**	**98.23**

Abbreviations: G, guava; Gr, grapes; W, watermelon.

### 4.3 Robust Analysis With Multiple Seeds

While the proposed model demonstrates superior performance compared to baseline approaches, it is equally important to evaluate its robustness under different random initializations. Therefore, we perform a multiseed analysis by training and testing the model using five distinct random seeds across three datasets: watermelon, grapes, and guava. The results, summarized in Table 7, indicate that the proposed model maintains highly stable and consistent performance across different runs. For the watermelon dataset, the model achieves an average accuracy of 99.74% with a very low standard deviation of ±0.0032, along with similarly stable precision, recall, and F1‐score values. A comparable trend is observed for the grapes dataset, where the model attains an average accuracy of 99.76% (±0.0029), demonstrating near‐perfect and consistent predictions for all seeds. Although slightly higher variability is observed in the guava dataset, the model still maintains strong performance with an average accuracy of 97.59% (±0.0086), indicating robust generalization. Overall, the low standard deviation across all evaluation metrics confirms that the proposed model is not sensitive to random initialization and produces reliable and reproducible results across different datasets.

**TABLE 7:** Robustness analysis of proposed model across multiple random seeds.

Dataset	Seed	Accuracy (%)	Precision (%)	Recall (%)	F1‐score (%)
Watermelon	42	99.86	99.89	99.90	99.90
	123	100	100	100	100
	999	100	100	100	100
	2024	99.86	99.89	99.90	99.90
	7	99.57	99.52	99.52	99.52
	**Mean ± std.**	**99.74 ± 0.0032**	**99.43 ± 0.0058**	**99.42 ± 0.0059**	**99.42 ± 0.0059**
Grapes	42	100	100	100	100
	123	99.80	99.81	99.79	99.80
	999	100	100	100	100
	2024	100	100	100	100
	7	99.01	99.98	99.03	99.01
	**Mean ± std.**	**99.76 ± 0.0029**	**99.76 ± 0.0039**	**99.76 ± 0.0039**	**99.76 ± 0.0039**
Guava	42	96.19	96.35	96.02	96.15
	123	96.82	97.26	96.78	96.96
	999	98.73	98.70	98.79	98.74
	2024	95.68	95.68	95.85	95.73
	7	97.33	97.39	97.29	97.30
	**Mean ± std.**	**97.59 ± 0.0086**	**97.02 ± 0.0103**	**96.95 ± 0.0105**	**96.95 ± 0.0105**

### 4.4 Benchmark

Table 8 provides a comparative analysis of existing leaf disease detection models in relation to the proposed LeafXNet architecture, evaluated on three fruit leaf datasets: watermelon, grapes, and guava. The evaluation highlighted interpretability, model complexity (measured by the number of parameters), and classification accuracy. We observed that only a limited number of existing studies have emphasized model interpretability, although this is crucial for effective decision‐making in real‐world agricultural applications. Across all datasets, LeafXNet demonstrated superior performance, consistently achieving higher accuracy with substantially fewer parameters compared to existing approaches. Notably, a minor variation in parameter count was observed: The watermelon and grape datasets each required 79,588 parameters, whereas the guava dataset required 79,717 parameters. This difference is attributed to the number of output classes, with watermelons and grapes consisting of four classes each, whereas guava includes five classes. Unlike prior models, which often sacrifice accuracy for reduced complexity or achieve higher accuracy at the cost of massive parameter sizes, LeafXNet effectively balances both dimensions. Our model surpasses existing methods by offering a drastic reduction in computational requirements while simultaneously delivering state‐of‐the‐art accuracy. This clearly demonstrates that LeafXNet not only challenges but also outperforms conventional approaches across multiple fruit leaf disease detection tasks.

**TABLE 8:** Comparison of existing leaf disease models with the proposed LeafXNet model in terms of interpretability, model complexity, and accuracy.

Fruit	Name	Year	Method used	Dataset size	Interpretability	Parameters	Accuracy
Watermelon	Islam et al. (2024)	2024	Dense‐EfficientNet	1115	✓	15.3 M	98.40
	Tas et al. (2025)	2025	VGG19	1500	×	143.7 M	97.78
	Madadum et al. (2025)	2025	YOLOv11n	2076	×	2.58 M	89.00
	Dharmawan (2025)	2025	LeNet‐5	1155	×	60,850	93.00
	Shikdar et al. (2024)	2024	CNN	5775	✓	3.9 M	99.68
	**Proposed**	**2026**	**LeafXNet**	**6930**	✓	**79,588**	**99.74 ± 0.0032**
Grapes	Lin et al. (2022)	2022	GrapeNet	2850	✓	2.15 M	86.29
	Wang et al. (2022)	2022	CNN	4062	×	0.72 M	96.70
	Nargotra and Sharma (2023)	2023	CNN	5500	×	44.4 M	90.44
	Prathiksha et al. (2024)	2024	VGG19	2980	×	143.7 M	98.00
	Chen et al. (2024)	2024	YOLOv8‐ACCW	10,000	×	2.8 M	92.80
	Dharrao et al. (2025)	2025	ResNet‐18	2726	×	11.7 M	96.00
	**Proposed**	**2026**	**LeafXNet**	**5016**	✓	**79,588**	**99.76 ± 0.0029**
Guava	Mustak Un Nobi et al. (2023) Doutoum et al. (2023)	2023 2023	GLD‐Det EfficientNet‐B3	5426 1894	× ✓	9 M 12.3 M	97.14 94.93
	Foo et al. (2024)	2024	InceptionV3	500	×	21.8 M	85.00
	Thangaraj et al. (2023)	2023	DenseNet169	2300	×	14.3	96.12
	Güler et al. (2025)	2025	Ensemble DL	2063	×	182 M	97.50
	Karthikeyan et al. (2025)	2025	GuavaCareNet	8000	×	12 M	98.32
	**Proposed**	**2026**	**LeafXNet**	**7856**	✓	**79,717**	**99.74 ± 0.0032**

### 4.5 Computational Complexity

Table 9 presents a comparison of the computational complexity of the proposed LeafXNet model with a selected subset of state‐of‐the‐art architectures, including XCEPTION, DenseNet121, InceptionV3, and NasNetMobile. The proposed model demonstrates a highly lightweight design, requiring only 79,717 parameters and 0.30 MB of storage, which is significantly lower than all compared models. In terms of computational cost, LeafXNet achieves 1.05 GFLOPs, indicating a reduced number of operations compared to deeper networks such as InceptionV3 and XCEPTION. Furthermore, the latency analysis shows that LeafXNet consistently achieves the lowest inference time across all datasets, particularly outperforming the baseline models in the Guava class. Although some models exhibit competitive latency in certain cases, they do so at the expense of higher computational complexity and larger model size. Overall, the proposed LeafXNet achieves an effective balance between compactness and inference speed, making it more suitable for real‐time and resource‐constrained applications.

**TABLE 9:** Comparison of computational complexity of the proposed model with selected state‐of‐the‐art baseline models.

Model	Parameters	Model size (MB)	FLOPs (GFLOPs)	Latency (ms)		
				W	Gr	G
XCEPTION	21,124,397	80.58	9.13	49.17	6.53	57.56
DenseNet121	7,169,349	27.35	5.70	50.97	6.72	56.76
InceptionV3	22,065,701	84.17	11.47	9.82	7.43	55.32
NasNetMobile	4,405,657	16.81	1.15	9.94	6.35	57.55
**LeafXNet**	**79,717**	**0.30**	**1.05**	**7.75**	**5.85**	**9.55**

### 4.6 Insights From XAI

A comparative visualization of CAM, Grad‐CAM, and Grad‐CAM++ for four classes in a watermelon disease classification task: downy mildew, anthracnose, mosaic virus, and healthy, is exhibited in Figure 6. CAM induces broad activation across the leaf surface in the downy mildew class, but lacks specificity. Grad‐CAM, on the other hand, concentrates on angular yellow lesions near the leaf edges, which are typical sites of disease initiation. This is further refined by Grad‐CAM++, which sharply localizes the chlorotic regions along the margins and near the vessels, providing biologically meaningful interpretations that are consistent with disease progression. CAM emphasizes fruits in the anthracnose class, albeit with restricted precision. Grad‐CAM enhances the focus on the central necrotic spot, whereas Grad‐CAM++ precisely depicts the diseased area with a high degree of focus on the concentric ring‐like lesion and surrounding tissue discoloration, which are critical visual indicators of fungal infection. The mosaic virus exhibits weak disease localization, as CAM broadly activates random patches across the leaf. Grad‐CAM identifies certain regions with vein clearing and pattern distortion, whereas Grad‐CAM++ distinctly emphasizes mosaic‐like mottling and irregular pigmentation patterns across the leaf surface hallmarks of viral infection by disregarding healthy tissue. CAM and Grad‐CAM generate inconsistent and dispersed activations in the healthy class, which may result in misinterpretation. In contrast, Grad‐CAM++ effectively suppresses attention throughout the entire image, suggesting that the model does not rely on any anomalous regions to perform the classification. Consequently, confidence in the “Healthy” prediction is increased. Collectively, these findings indicate that Grad‐CAM++ consistently offers the most precise and symptom‐aware visual explanations across all disease categories, thereby substantially improving the transparency and trustworthiness of the model in real‐world agricultural diagnostics. To further validate the biological relevance of these highlighted regions, Dr. Mohammad Idris Ali Howladar, Principal Scientific Officer, Regional Horticulture Research Station, Lebukhali, Patuakhali, manually examined and annotated a representative subset of images.

**FIGURE 6:**
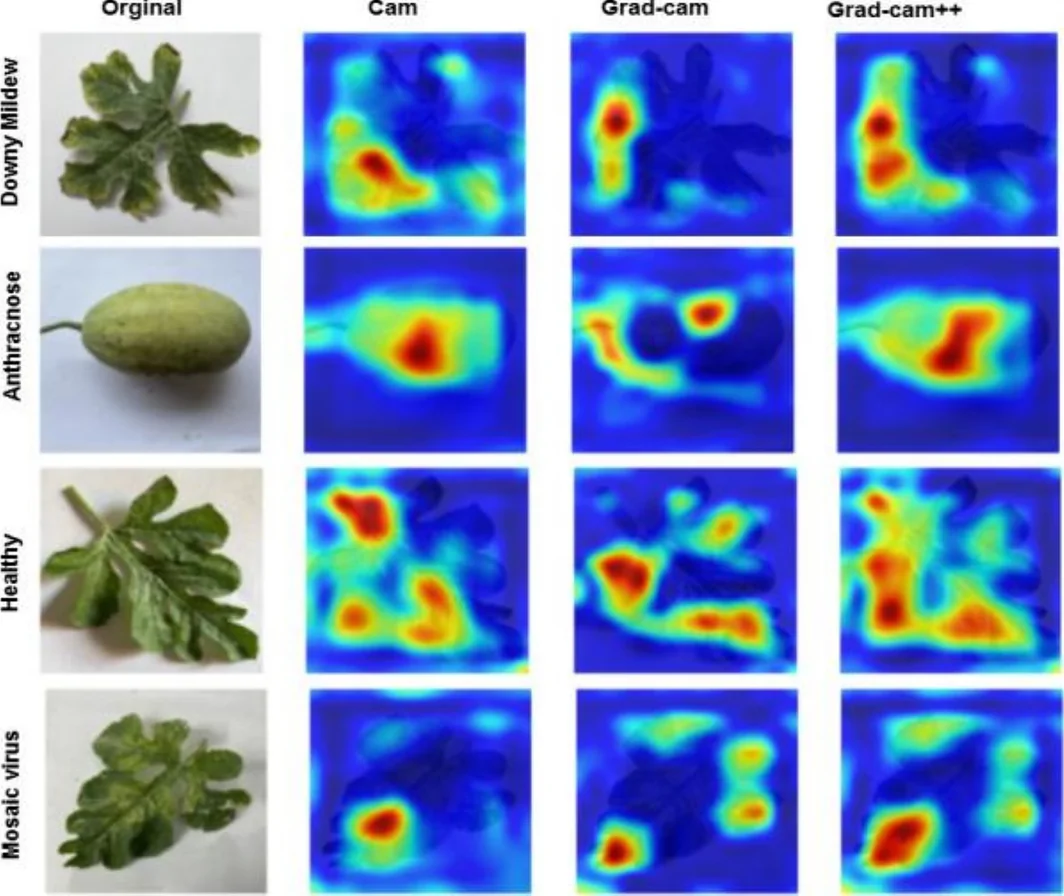
Explainable AI visualization of watermelon leaf disease patterns.

The XAI visualizations for grape leaf classification using CAM, Grad‐CAM, and Grad‐CAM++ across the four conditions are shown in Figure 7. Grad‐CAM concentrates on the upper lesion areas, whereas CAM produces dispersed highlights across the leaf surface in the case of bacterial leaf spot. Grad‐CAM++ further narrowed the focus, isolating the small irregularly shaped spots along the infected margin. The infected lower region was more clearly captured by Grad‐CAM, and Grad‐CAM++ further emphasized this by emphasizing two distinct clusters of infection. For downy mildew, CAM exhibits activation near the central part of the leaf but spreads beyond the lesion boundary. Despite the absence of disease, CAM and Grad‐CAM both exhibited scattered activation on the healthy leaf. Grad‐CAM++, on the other hand, eliminates superfluous highlights, resulting in only mild activation in noncritical regions. Grad‐CAM identifies the lower diseased area with a stronger intensity, whereas Grad‐CAM++ delineates the characteristic mildew patches more distinctly, highlighting concentrated regions of infection while suppressing irrelevant areas, for powdery mildew. However, CAM distributes attention broadly across the leaf.

**FIGURE 7:**
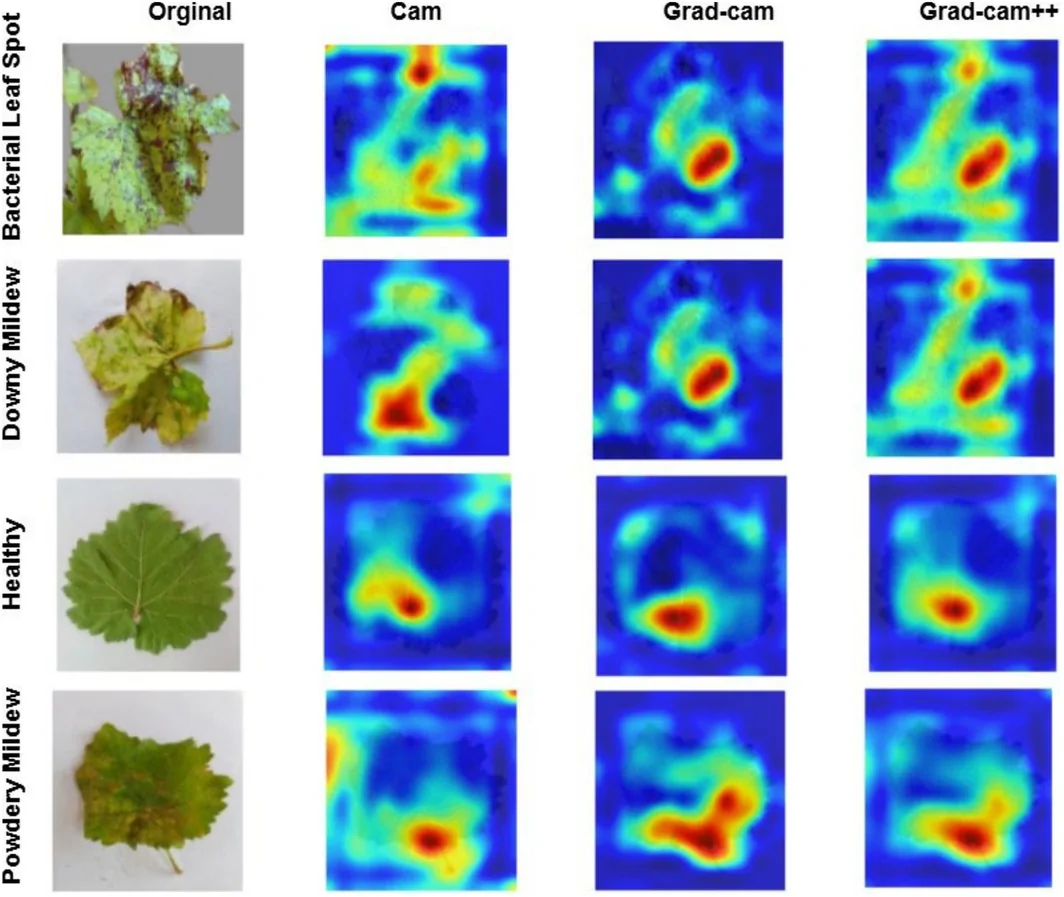
Explainable AI visualization of Grapes leaf disease patterns.

The CAM, Grad‐CAM, and Grad‐CAM++ visualizations for guava leaf classification across the anthracnose, canker, dot, healthy, and rust categories are shown in Figure 8. In anthracnose, CAM generates extensive activations throughout the leaf, whereas Grad‐CAM emphasizes the lesion along the margin. Grad‐CAM++ further refines this by isolating the irregular necrotic areas. Grad‐CAM begins to capture the darker infected boundary, whereas Grad‐CAM++ distinctly defines the localized infection near the leaf edge.

**FIGURE 8:**
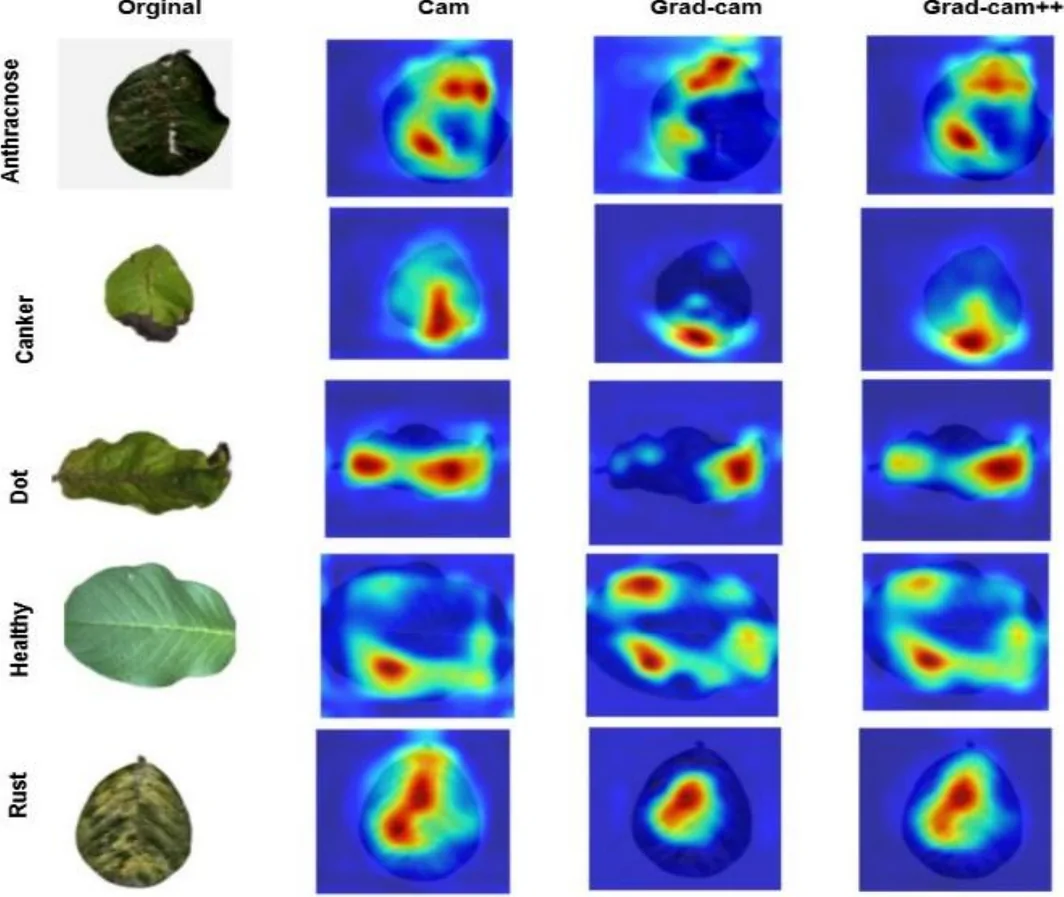
Explainable AI visualization of grapes leaf disease patterns.

For canker, CAM displays a general focus in the central region. Grad‐CAM and CAM have spread attention over extensive portions of the leaf in dot disease. However, Grad‐CAM++ narrows the focus, correctly emphasizing the small scattered dot‐like lesions. Grad‐CAM++ diminishes extraneous attention, suggesting the absence of disease‐specific patterns, whereas CAM and Grad‐CAM continue to exhibit multiple activations in distinct zones in healthy leaf. Lastly, CAM generates a central activation for rust, Grad‐CAM enhances the focus over the lesion cluster, and Grad‐CAM++ accomplishes the most precise localization by clearly delineating the rust patches along the mid and side veins. Grad‐CAM++ consistently exhibited activations that were more symptom‐specific and sharper across all conditions, which is more consistent with the visual evidence in the original leaf images.

### 4.7 Quantitative Expert‐Based Validation of XAI Explanations

To complement the qualitative observations illustrated in Figures 6, 7, 8, a quantitative expert‐based assessment was performed to systematically evaluate the quality and interpretability of the generated explanations. In total, 300 leaf images were assessed across the three datasets, including 100 images from the watermelon dataset, 100 images from the grape dataset, and 100 images from the guava dataset, with 25 images selected from each disease class. For every selected image, the explanation maps produced by CAM, Grad‐CAM, and Grad‐CAM++ were independently examined by Dr. Mohammad Idris Ali Howladar, Principal Scientific Officer, Regional Horticulture Research Station, Lebukhali, Patuakhali. Drawing on his professional expertise, he recommended three assessment dimensions, namely, morphological relevance, localization precision, and feature correspondence, and provided guidance regarding their interpretation throughout the evaluation process. Morphological relevance evaluates whether the regions emphasized by an explanation correspond to visually meaningful morphological patterns and disease‐related symptoms that are relevant to the diagnosis of the respective plant disease. Localization precision assesses the extent to which the highlighted regions accurately identify the specific portions of the leaf exhibiting visible disease symptoms or abnormal characteristics. Feature correspondence examines whether the activated regions are consistent with the visual features that are considered informative for recognizing and differentiating the corresponding plant diseases. Each dimension was scored using a 5‐point rating scale, where higher values indicate stronger agreement between the highlighted regions and disease‐relevant visual or morphological characteristics.

The quantitative findings are presented in Table 10, with results reported as mean ± standard deviation. The mean score represents the average expert rating assigned to each evaluation criterion across the assessed images, whereas the standard deviation reflects the degree of variation in the expert assessments. Consequently, a lower standard deviation indicates greater consistency in the assigned ratings. The overall mean score was calculated by averaging the scores obtained across the three evaluation criteria. Higher overall scores therefore indicate explanations that more consistently emphasize visually meaningful and diagnostically relevant regions associated with plant disease characteristics.

**TABLE 10:** Expert‐based assessment of XAI methods using three interpretability criteria.

XAI method	Morphological relevance	Localization precision	Feature correspondence	Overall mean
CAM	3.18 ± 0.69	3.06 ± 0.67	3.17 ± 0.71	3.13
Grad‐CAM	3.80 ± 0.58	3.87 ± 0.59	3.90 ± 0.59	3.86
**Grad‐CAM++**	**4.47 ± 0.56**	**4.42 ± 0.59**	**4.43 ± 0.59**	**4.44**

The results demonstrate a clear difference in explanation quality among the three interpretation methods. Grad‐CAM++ achieved the strongest performance across all evaluation dimensions, with scores of 4.47 ± 0.56 for morphological relevance, 4.42 ± 0.59 for localization precision, and 4.43 ± 0.59 for feature correspondence. Grad‐CAM produced intermediate results, recording 3.80 ± 0.58, 3.87 ± 0.59, and 3.90 ± 0.59 for the corresponding evaluation criteria. In comparison, CAM obtained comparatively lower scores of 3.18 ± 0.69, 3.06 ± 0.67, and 3.17 ± 0.71. The resulting overall mean scores were 4.44 for Grad‐CAM++, 3.86 for Grad‐CAM, and 3.13 for CAM. These findings indicate that Grad‐CAM++ more consistently directs attention toward disease‐relevant regions of the leaves and produces explanations that are more closely aligned with visually distinguishable symptoms and morphological characteristics. In contrast, Grad‐CAM provides moderately effective visual explanations, whereas CAM exhibits comparatively lower agreement with expert assessments across all evaluation criteria.

To determine whether the differences observed among the three explanation methods were statistically meaningful, a nonparametric Friedman test was conducted separately for each evaluation criterion using the 300 paired image‐level assessments. This test was selected because it is appropriate for comparing more than two related methods without requiring the assumptions associated with parametric statistical procedures. The analysis examined whether the expert ratings differed significantly among CAM, Grad‐CAM, and Grad‐CAM++ for morphological relevance, localization precision, and feature correspondence. As reported in Table 11, statistically significant differences were identified for all three criteria, including morphological relevance (χ^2^ = 389.77, *p* < 0.001), localization precision (χ^2^ = 391.78, *p* < 0.001), and feature correspondence (χ^2^ = 378.53, *p* < 0.001). These findings indicate that the expert ratings differed significantly among the three explanation methods across all evaluation criteria, confirming that CAM, Grad‐CAM, and Grad‐CAM++ did not perform equivalently in terms of the quality and disease relevance of their visual explanations.

**TABLE 11:** Comparison of XAI methods using the Friedman test across three expert evaluation criteria.

Criterion	Friedman χ^2^	*p*	Result
Morphological relevance	389.77	< 0.001	Significant
Localization precision	391.78	< 0.001	Significant
Feature correspondence	378.53	< 0.001	Significant

Because the Friedman test establishes the presence of an overall difference among the methods but does not identify the specific method pairs responsible for that difference, post hoc pairwise comparisons were subsequently conducted using the Wilcoxon signed‐rank test with Bonferroni correction. The Wilcoxon signed‐rank test was employed as a nonparametric paired comparison procedure to assess differences between the expert ratings assigned to each pair of explanation methods. For each comparison, the paired differences between the corresponding ratings were ranked according to their absolute magnitude, and the resulting rank statistics were used to determine statistical significance. To account for the increased probability of false‐positive findings resulting from multiple pairwise comparisons, Bonferroni correction was applied to the resulting *p*‐values.

The results of the post hoc analysis, summarized in Table 12, revealed statistically significant differences for all pairwise comparisons across the three evaluation criteria after Bonferroni adjustment. For morphological relevance, significant differences were observed between CAM and Grad‐CAM (Wilcoxon statistic = 1976.5, adjusted *p* = 1.11 × 10^−27^), CAM and Grad‐CAM++ (938.0, adjusted *p* = 2.87 × 10^−45^), and Grad‐CAM and Grad‐CAM++ (751.5, adjusted *p* = 1.38 × 10^−33^). Similarly, for localization precision, all pairwise comparisons were statistically significant, including CAM versus Grad‐CAM (1165.5, adjusted *p* = 1.22 × 10^−34^), CAM versus Grad‐CAM++ (1338.0, adjusted *p* = 1.16 × 10^−43^), and Grad‐CAM versus Grad‐CAM++ (886.0, adjusted *p* = 5.50 × 10^−29^). For feature correspondence, significant differences were likewise identified between CAM and Grad‐CAM (1499.0, adjusted *p* = 1.04 × 10^−31^), CAM and Grad‐CAM++ (824.0, adjusted *p* = 1.98 × 10^−45^), and Grad‐CAM versus Grad‐CAM++ (1501.0, adjusted *p* = 4.97 × 10^−26^). These findings demonstrate that each explanation method differs significantly from the others across all expert evaluation criteria.

**TABLE 12:** Post hoc pairwise comparisons of XAI methods using the Wilcoxon signed‐rank test with Bonferroni correction.

Criterion	Comparison	*N*	Wilcoxon statistic	Adjusted *p*‐value
Morphological relevance	CAM vs. Grad‐CAM	300	1976.5	1.11 *×* 10^−27^
	CAM vs. Grad‐CAM++	300	938.0	2.87 *×* 10^−45^
	Grad‐CAM vs. Grad‐CAM++	300	751.5	1.38 *×* 10^−33^
Localization precision	CAM vs. Grad‐CAM	300	1165.5	1.22 *×* 10^−34^
	CAM vs. Grad‐CAM++	300	1338.0	1.16 *×* 10^−43^
	Grad‐CAM vs. Grad‐CAM++	300	886.0	5.50 *×* 10^−29^
Feature correspondence	CAM vs. Grad‐CAM	300	1499.0	1.04 *×* 10^−31^
	CAM vs. Grad‐CAM++	300	824.0	1.98 *×* 10^−45^
	Grad‐CAM vs. Grad‐CAM++	300	1501.0	4.97 *×* 10^−26^

Taken together, the descriptive and inferential statistical analyses establish a consistent performance hierarchy of Grad‐CAM++ > Grad‐CAM > CAM across morphological relevance, localization precision, and feature correspondence. Grad‐CAM++ obtained the highest expert ratings and demonstrated the strongest correspondence with disease‐related visual characteristics, whereas Grad‐CAM showed intermediate performance, and CAM received the lowest scores. These quantitative findings reinforce the qualitative observations presented in Figures 6, 7, 8, indicating that Grad‐CAM++ produces explanations that are comparatively more focused on disease‐relevant regions and more precisely aligned with the morphological characteristics of symptomatic plant leaves. The results therefore provide robust quantitative expert‐based evidence supporting the superior interpretability of Grad‐CAM++ for plant leaf disease classification.

## 5 Conclusion

To identify fruit leaf disease accurately and efficiently, we developed a lightweight, attention‐fused CNN called LeafXNet. The model's great generalization capacity across numerous fruit types was proved by testing it on three different datasets: watermelon, grape, and guava. The accuracies obtained were 99.74 ± 0.0032, 99.76 ± 0.0029, and 97.59 ± 0.0086, respectively. With only 79,717 parameters, LeafXNet drastically lowers the computational overhead, which makes it ideal for deployment on web‐based apps, mobile platforms, and IoT devices. Our use of XAI approaches improves model transparency and increases user confidence, addressing one of the main barriers to the practical adoption of deep learning models, which are frequently black boxes. This is further confirmed by comparison with current state‐of‐the‐art techniques in that LeafXNet performs better than intricate pretrained CNNs while using fewer resources. Overall, this study highlights the potential of lightweight and interpretable deep learning models to advance sustainable agriculture by enabling the early and reliable detection of plant diseases. An important limitation is that this study focuses on disease‐versus‐healthy leaf classification and does not distinguish diseases from visually similar nondisease physiological disorders; therefore, LeafXNet should be used as a decision‐support tool rather than a standalone diagnostic system. Future work may include expanding LeafXNet to include more crops, including real‐time monitoring systems, and creating farmer‐friendly applications that better connect AI research with practical agricultural methods. In addition, evaluating the model's transferability to unseen species remains an important direction for future investigation.

## Author Contributions

**Shah Murtaza Rashid Al Masud:** visualization, validation, formal analysis, conceptualization. **Farhana Sultana Eshita:** writing original draft, formal analysis, methodology, conceptualization. **Abu Bakar Siddique Mahi:** methodology, investigation, formal analysis, conceptualization, visualization, validation, writing original draft, supervision. **Tasnim Jahin Mowla:** writing – original draft. **Noor Mairukh Khan Arnob:** writing review and editing, visualization, validation, supervision. **Aloke Kumar Saha:** writing review and editing, visualization, validation, supervision. **M.F. Mridha:** writing review and editing, visualization, validation, supervision.

## Funding

This work is supported by the Institute of Energy, Environment, Research, and Development (IRID), University of Asia Pacific (UAP), Bangladesh.

## Conflicts of Interest

The authors declare no conflicts of interest.

## Data Availability

All datasets are publicly available here: **Watermelon:** https://data.mendeley.com/datasets/ntzym554jp/1 **Grapes:** https://data.mendeley.com/datasets/8nnd2ypcv3/5 **Guava:** https://data.mendeley.com/datasets/fspx44mwfp/.
